# Photographs of Actions: What Makes Them Special Cues to Social Perception

**DOI:** 10.3390/brainsci11111382

**Published:** 2021-10-22

**Authors:** Leopold Kislinger

**Affiliations:** Independent Researcher, Cranachstraße 39/3, 4060 Leonding, Austria; leopold.kislinger@ufg.at

**Keywords:** photography, action photographs, action observation network, mirror neurons, transcranial magnetic stimulation (TMS), corticospinal excitability (CSE), pictorial representation

## Abstract

I have reviewed studies on neural responses to pictured actions in the action observation network (AON) and the cognitive functions of these responses. Based on this review, I have analyzed the specific representational characteristics of action photographs. There has been consensus that AON responses provide viewers with knowledge of observed or pictured actions, but there has been controversy about the properties of this knowledge. Is this knowledge causally provided by AON activities or is it dependent on conceptual processing? What elements of actions does it refer to, and how generalized or specific is it? The answers to these questions have come from studies that used transcranial magnetic stimulation (TMS) to stimulate motor or somatosensory cortices. In conjunction with electromyography (EMG), TMS allows researchers to examine changes of the excitability in the corticospinal tract and muscles of people viewing pictured actions. The timing of these changes and muscle specificity enable inferences to be drawn about the cognitive products of processing pictured actions in the AON. Based on a review of studies using TMS and other neuroscience methods, I have proposed a novel hypothetical account that describes the characteristics of action photographs that make them effective cues to social perception. This account includes predictions that can be tested experimentally.

## 1. Photographs of Actions: What Makes Them Special Cues to Social Perception

I will introduce action photographs (henceforth, photos) as visual cues that evoke neural activations in the action observation network (AON) of viewers and address controversial explanations of the cognitive products of these activations. In the first section, I will briefly define two concepts that play a central role in many of the studies reviewed in this paper: action representation and knowledge of actions. Then, I will give an overview of findings on the cognitive products of the activations in the AON, coming primarily from studies in which live actions or video clips of actions were used as stimuli. Next, I will address the cognitive responses to action photos and the particular social perception that they convey. In the following section, I will analyze the particular representational characteristics of action photos that are assumed to be the most relevant to this particular perception. I will illustrate these characteristics with some picture examples, in which action photos have been modified so that only the picture information which is relevant with regard to the processing in the AON in the first 300 milliseconds after picture onset could be seen. I will conclude by addressing questions for future research.

The present review is based on the following assumptions: Action photos convey to viewers a similar particular social perception as observing live actions. This social perception is based on neural activities in the AON and physiological excitation states in the body outside the brain. Within a few hundred milliseconds of processing, seeing action photos conveys to viewers a specific motor and somatosensory knowledge. Only a part of the components or elements of the actions depicted are relevant with regards to this knowledge. To examine these assumptions, I have reviewed studies on the AON that provides relevant information.

The AON is a network of regions in the occipito-temporal, temporal, parietal, and prefrontal cortices [1,2]. Neural responses to action photos in the AON have been observed in many studies (for references, see Table 1; this table also takes into account emotional areas or centers that are involved in processing pictured actions). The AON includes the brain regions that contain mirror neurons [1,3]. Researchers first discovered mirror neurons in the premotor cortex of macaque monkeys [4,5]. They are a type of visuomotor neuron, which are activated when monkeys see another individual performing a certain action and when they perform this action themselves. Mirror neurons have also been found in parietal regions; the different regions that contain mirror neurons together form the mirror neuron system [3]. Analogous neurons and mechanisms were subsequently also found in humans who observed live or videoed actions [6]. For a review, see [7]. Mirror neurons “vicariously” build the motor activation patterns that are currently present in the motor system of the observed individual [8]. Vicarious neural activations associated with somatosensory components of actions have been observed in somatosensory cortices and the insula [9,10,11]. The AON also includes brain areas that do not contain mirror neurons but are nevertheless specifically involved in the processing of action-related visual information [10,12,13] (see also Table 1).

There is a broad consensus in the research literature that the processing of observed live actions in the AON provides observers with knowledge of what others are doing and that this is a key skill of social cognition [8,23,24,25,38,42,43]. There is, however, controversy about the properties of the knowledge conveyed by neural AON activities: is this knowledge only provided by AON activities, or is it dependent on conceptual and semantic processing [15,44,45,46,47,48,49]? Is it primarily related to understanding the goal of actions [3,5,15], or does it relate equally to several elements of actions, such as grips, movements of body parts, somatosensory processes, objects involved, or context [50,51,52]? A key issue is how generalized or specific the action-related information is that comprises this knowledge [7,47,50].

This question is also relevant with regards to action photos. Do the AON responses convey knowledge of the specific properties of the concrete pictured actions [2,8,13,36,53,54,55,56], or do they provide an abstract understanding based on categorization processes [3,5,15]? Decisive answers to this question come from studies that have used the technique of transcranial magnetic stimulation (TMS) to modulate the neural activity of specific brain areas (Table 2). TMS studies suggest that AON responses to action photos represent specific properties and elements of the actions depicted [36,55,56,57,58,59,60,61,62,63]. Studies in which TMS was used in conjunction with electromyography (EMG) also provide findings about the time course of AON responses to action photos (Table 2). Data on the timing of these responses are of central importance for the delimitation of the picture-related perceptual processes from other cognitive operations that are also related to AON activations. Such cognitive operations, for example, are drawing inferences from action-related picture information [55,64,65,66] or voluntary activities, like mentalizing [64,67] and motor imagery [68,69,70].

To the best of my knowledge, no hypothetical account has been proposed to date that has identified the representational core characteristics of action photos and the specific perception they convey. Based on a review of TMS studies on AON responses as well as investigations using a wide range of neuroscientific methods, the present article intends to fill this gap. The proposed characteristics of action photos and cognitive processes correspond to predictions that can be tested experimentally.

## 2. Definitions of Key Terms and Concepts

In this section, I will briefly define key terms and concepts that many researchers use or refer to when studying the cognitive functions of neural activity in the AON. The first subsection describes properties of neural representations that are relevant with regards to the particular perception conveyed by action photos. The second subsection describes the knowledge of actions that is represented by fast AON responses to action photos and the key elements that this knowledge encompasses.

### 2.1. Neural Representations of Actions

Little is known about how motor actions are represented in the brain [78,79,80,81]. While performing a goal-directed action, pieces of the processed information are stored in the motor, somatosensory, visual, corticospinal, and muscular system of the acting individual for a short time [79,80]. Information stored in the brain, called memory content here, is allocated to a neural structure (specific neurons, synapses, or neural circuits) through special mechanisms [82]. Allocation mechanisms determine the locations at which memory content is stored and how much storage space is allocated to it [82,83].

According to the engram theory of memory [83,84], memories are stored in the brain by means of neural engrams. An engram is a specific pattern that is permanently carried by a specific population of neurons and represents a specific memory. When such an engram is activated, the memory that is carried by it is expressed or put into effect. Memory engrams can be composed of widely distributed neural ensembles [84]. In addition, information that is being processed is often assigned to populations of neurons in the brain that overlap. Memories about experiences that are organized in such a way that they share individual components with other memories are interconnected and organized within associative networks [85].

Representations of actions are presumably carried by neural engrams that span widely and are distributed in cortical and subcortical regions [78]. Cortical sensorimotor, premotor, and associative regions (cortical motor, pre-motor, and association regions); cerebellar sensorimotor regions; basal ganglia; and the spinal cord are likely involved [78,79]. In relation to how generalized or specific the knowledge is that individuals associate with observed actions, it may be relevant that memory engrams are functionally heterogeneous. They contain neural ensembles for both memory discrimination and memory generalization [86]. A memory engram that represents a motor action, therefore, could include neural ensembles that support generalization operations and neural ensembles that support specification. In this case, motor and somatosensory knowledge that is expressed by neural activations in the AON would have a certain degree of specificity, which could be indicated along a continuous scale. At one end of this scale are abstract categories of actions or action elements; at the other end are rich sensorimotor memories that relate to specific actions experiences.

### 2.2. Knowledge of Actions

The word knowledge means “to be in possession of information” [87] (p. 148). The information that is known can relate to different modes, such as concepts, categories, words, movements, emotions, somatosensory processes, or physiological excitation states [88]. Knowledge enables individuals to “act on the information known” [87] (p. 149).

The processing of visual information by the AON provides people with knowledge of actions being seen (for review, see [3,7]). According to one influential view, this knowledge is the result of a fast automatic “transformation” [3] (p. 655) of the received visual information into a motor representation of an action. The ability to perform this transformation has developed over the course of evolution, because this ability was advantageous for individuals living together in groups and supported fast adaptive social behavior. According to this view, observers come into possession of knowledge about an observed action on the basis of evolutionarily inherited brain mechanisms that work automatically. Knowledge of actions can also result from individual past experiences. In this case, the special properties of the neurons of the AON and mirror neuron systems are the result of individual associative learning [60,70,89]. It allows individuals to associate an observed action with a certain action-related knowledge. If people see an action that they are not familiar with, they can still recognize and understand it if it comprises components that are available in the viewer’s motor or somatosensory memory [90].

The knowledge of actions comprises neural or mental [88] representations of various action components or elements. The literature on action representations suggests that recognizing and understanding observed actions involves six key elements:

1. Movements of body parts and the spatial and temporal properties of these movements, such as distance, direction or trajectory, speed, acceleration, or duration [50,91,92].
2. Internal models for the control of the muscle activities that generate the movements. Various scholars have described the control of actions by signals from the brain using motor programs [93] or on the basis of models in which individuals select motor commands for a specific context, depending on multiple internal and external factors [92]. Motor programs are representations of rules for the execution of movements, according to which the spatial and temporal activity patterns of certain muscles are organized and controlled [93]. These programs are supposed to be stored in motor brain structures in a generalized or abstract format. “Models for motor control” [94], on the other hand, describe the control of actions more in connection with adjustments to specific action contexts and courses. Individuals transform sensory information into motor commands. The resulting movements produce sensory outcomes that provide feedback for further motor control.
3. Somatosensory processes or sensations, for example, in relation to proprioception, the processing of haptic or tactile information, heat, cold, or pain [9,10,74,92,95,96]. A crucial property of the knowledge about movements, internal models, and somatosensory processes and sensations is that this knowledge includes information about changes over time and outcomes of these changes [10,56,92,94]. Individuals can use this change-related information to anticipate the immediate further course of an action that they are performing or observing [56,62,73,97]. Somatosensory anticipation plays a particularly important role in performing actions [10,79,96]. It conveys information about the immediate somatosensory consequences of movements, for example, proprioceptive or tactile stimulation.
4. Objects and contexts associated with actions [92,94]. Actions are often directed towards objects or include the use of objects, for example, food, clothing, tools, vessels with drinks, or weapons [98,99,100].
5. Knowledge of the desired outcomes of movements, that is, of action goals [91]. Knowledge of goals includes goals at different hierarchical levels. The overarching goal of an action is often referred to in the literature as the “intention” [3,50]. Goals of actions are related to motives, needs, or desires and have a certain importance or value. For this reason, mental representations of motor actions fundamentally include an emotional component [11,55,57].
6. Knowledge of the relevance or emotional value of actions or contexts of actions [11,55]. The term emotion refers to a response to an object or event that is important to individuals and requires them to prepare for an appropriate action [88,101].

## 3. Cognitive Products of Processing Observed Actions in the AON

Before I address findings on the specific cognitive operations that take place in viewers of action photos, I will give a brief overview of explanations of the cognitive correlates or products of the neural activities in the AON, coming primarily from studies in which live actions or video clips of actions were used as stimuli. These studies suggest five cognitive products: (1) action understanding, (2) knowledge of specific properties of the observed actions, (3) changes in motor and somatosensory excitability, (4) activation of a motivational or emotional state, and (5) experiences that are accompanied by conscious awareness.

The word “cognitive” is used in a broad sense here. It denotes processes in the nervous system that are related to the use of information for the selection of adaptive behavior or problem solving. What is generated through cognitive processes is meaning. The term “cognitive function” describes a specific contribution of neural information processing to the well-being, prosperity, survival, or reproductive success of individuals [102].

### 3.1. Action Understanding

Research on the neural processing of visual information about actions has focused on understanding [3,50,74,103,104]. Understanding actions primarily relates to gaining knowledge about the goals and intentions underlying the observed movements [3,5,15]. An observer, for example, sees another individual grasping an apple and understands that the individual wants to eat the apple. Regarding the timing of processing, the findings suggest that an observed grip is associated with an action goal, including information about an object involved or the action context, from around 250 ms after movement onset [46,105]. Processing in the extrastriate body area (EBA), middle temporal area (MT), and inferior parietal regions takes place in the time window of 120 to 200 ms after the movement onset [19,46,106,107,108].

#### 3.1.1. Generalization and Categorization

In explanations of AON responses that focus on action understanding, generalization and categorization play a central role [3,5,15]. In categorization, the quantity of information received is extremely minimized. The observer assigns the visual information to a specific group of action-related objects or events, like “catch”, “grasp”, “fight”, or “ball.” Such categories correspond to quick hypotheses about the basic meanings of pictured action elements and make corresponding action-related knowledge available [15,97,109].

Fast motor categorization is presumably based on signals from the magnocellular system. This is a special processing pathway from the retina to the cortex [45,110,111,112]. The magnocellular system processes information that has low spatial frequencies and is wavelength-insensitive [113,114,115]. The system is highly sensitive to contrast, has a low susceptibility to visual illusions, and is relatively fast. It fulfills an important function in the rapid localization of potentially relevant objects and movements in the visual field, which enables rapid motor behavioral responses [45,116,117]. The magnocellular pathway also projects into inferior parietal regions that belong to the AON and contain visuo-somatosensory neurons. Visuo-somatosensory neurons and neural ensembles in the somatosensory cortices could establish somatosensory categorization ([36]; for the review, see [51]). Regarding the topic of this review, it is important to note that the magnocellular system is also activated by seeing static pictures [116,117].

#### 3.1.2. Conceptual and Semantic Processes in Action Categorization

Generalization and categorization can be achieved by the activities of AON neurons with motor properties [5]. Such categorization is supported by the organization of the motor cortex into types of movement [3,118]. In the studies included in this review, however, action categorization is linked to conceptual and semantic processes. The researchers who discovered the mirror neurons associated these cells with the evolution and use of the human language from their first publication [4]. Rizzolatti and Craighero [104] spoke of the “semantics” (p. 184) of the mirror neuron system. Action categorization is assumed to involve interactions between the AON and areas of the ventral visual stream [43,44,45,48,49,50]. These interactions run via neural bi-directional connections that give the AON access to conceptual and semantic information. The ventral visual stream, in turn, gets access to action-related information [48].

Explanations of the cognitive products of the processes in the AON or the mirror neuron system in connection with semantic processes are questionable [51]. The activation of mirror neurons based on semantic processes requires that an observed action has already been recognized when the mirror neurons start to work. The explanation of the mirror neuron system as the neural mechanism that conveys the understanding of observed actions would thereby become circular [7].

The question of whether the processing in the AON causally provides specific knowledge about observed actions that is not conveyed through conceptual or semantic processes is difficult to investigate. The processing of conceptual action-related information in the ventral visual stream also leads to activations in the AON [65,119]. In addition, the AON responses to visual action-related information overlap with processes of motor imagery [68,69,70] and mentalizing [64,67], which also involve semantic processes. One group of findings on AON responses is particularly useful in clarifying the causal role of the AON in understanding actions: the specificity of the motor and somatosensory AON responses in relation to the concrete observed actions. I will refer to this in the next subsection.

### 3.2. Knowledge of Specific Properties of Observed Actions

Categorization involves generalization and a massive reduction of the information that is received and processed. Neural responses in the AON presumably also reflect the formation of motor and/or somatosensory activation patterns that represent specific properties of the observed actions more comprehensively than categories [8,36,44,50,54]. Specific properties of actions, for example, relate to properties of grips, movements, somatosensory activities, body postures, or objects. To my knowledge, there has been no report of a specific neural activation pattern in the AON that exactly and comprehensively expresses an entire concrete observed action. Two sets of evidence, however, suggest that representations of specific elements of observed actions are established in the AON.

The first set is related to features and functions of the brain areas included in the AON. The inferior parietal cortex and the ventral premotor cortex contain a high proportion of visual neurons, as well as many visuomotor and visuo-somatosensory neurons [5,6,42,120]. Such neurons reflect functions in the fast, accurate, and flexible visual guidance of actions in unique environments [121] and in the localization of possibly relevant individuals, movements, or objects in the visual field, which enables adaptive motor responses [43,45,116,117].

The second set of evidence is related to findings of studies in which the neural processes in certain areas of the AON were disturbed by TMS during action observation [10,12,54,63,64,73,74,122]. In this way, lesion-like effects were generated. These effects allowed researchers to investigate the specific contributions of cortical regions to the perception and understanding of actions, as well as causal links between these regions. The impairment of processes in somatosensory cortices provided decisive information about the fundamental role of the somatosensory cortices in the perception and understanding of observed actions [10,12,54,64,74]. Notably, lesion-like effects generated by TMS impaired the perception and recognition of specific properties of postures or movements of body parts [54,63] or of properties of the objects involved in actions [74]. These effects indicate that the neural representations of action elements that are established in the AON are more specific and comprehensive than would be the case with mere categorization. The formation of such action-specific neural representations takes a certain amount of time and begins around 150 ms after stimulus onset [7,26,59,66].

### 3.3. Changes in Motor and Somatosensory Excitability

Many researchers have used TMS in conjunction with electromyography (EMG) to investigate the effects of action observation on the excitability in the corticospinal tract and muscles (Table 2). For review, see also [7]. TMS, for example, has been used to stimulate the region of the primary motor cortex (M1), which is involved in preparing for a specific grasping movement. This stimulation leads to action potentials along the corticospinal pathway and generates larger or smaller motor-evoked potentials (MEPs) in muscles. The amplitude of the MEPs is measured transcutaneously using electromyography (EMG). It corresponds to the level of motor excitability [7].

If the MEPs are larger than in the baseline condition, this indicates an excitatory process. Action observation, in this case, results in a “facilitation” effect [72]. There is a muscle-specific modulation of the excitability when the MEPs, recorded from a muscle that was involved in an observed action, were changed during action observation, compared to the MEPs that were recorded during a baseline condition. In a systematic review of studies in which modulations of the corticospinal excitability were elicited by single pulse TMS, Naish and colleagues [7] found clear evidence of muscle-specific modulation in 16 of 24 studies. According to Naish et al., muscle specificity occurs from around 200 ms after the onset of observed movements; changes in excitability that occur earlier are not muscle-specific and are likely related to motivated visual selection or attention. When seeing emotionally charged actions, muscle specificity may occur earlier. In a study using TMS in conjunction with EMG, Borgomaneri and colleagues [55] found, in viewers of fearful body expressions, a selective reduction in excitability in a hand muscle involved in grasping. This reduction was measured 70–90 ms after stimulus onset and reflects a muscle-specific modulation of motor excitability when processing complex visual input in a very early time window.

An increase in muscle-specific excitability during action observation reflects cognitive functions related to the preparation or effective execution of observed movements, imitation [3,72], or empathy [58,77]. If the MEPs are smaller than in the baseline condition, the observation of the action is associated with an “inhibition” effect [7]. Inhibitory muscle-specific activities play a role in contexts in which movements are better not made [7,55]. This can be the case when it is advantageous for the observer to suppress an approach tendency, involuntary behavioral mimicry, or the imitation of an observed action.

Facilitation effects occur not only in muscles, but also in the muscle spindles, the proprioceptive receptors, that would be involved in actually performing the action [71]. In addition, seeing touch in connection with actions may modulate the excitability of skin receptors through descending projection trajectories from the somatosensory cortex [36]. Via upstream effects, the cortical representation of the proprioceptive elements of an observed action could thus have a physiological basis in the musculature and skin of the observer. Several researchers reported neural downstream projections into organs outside the brain that are related to the ability to react quickly and appropriately to action-related stimuli [55,56,62,73,123]. The AON may also code for the autonomic correlates of observed actions [124]. Observed actions can affect viewers’ cardiac activity [101,123,125,126,127]. Pictured physical exertion can be as effective or even more effective than the emotional value of a depicted action [128]. There are many findings on genital responses that are caused by pictures of sexual actions, for example, hemodynamic changes in the vaginal epithelium, changes in the skin temperature of the labia minora, or changes in penile erection [31,32,129].

### 3.4. Activation of a Motivational or Emotional State

Wanting to achieve a goal through body movements includes a motivational component. Facial expressions, as well as body and hand postures, may also provide emotional information to viewers [11,55,57,106,107]. In the brain responses of viewers, there are interactions between the processing of the motor, somatosensory, and emotional elements of observed behaviors. For a review, see [14,94]. The neural basis of the emotional processes involves the regions of the AON, as well as the amygdala, orbitofrontal cortex (OFC), insula, and anterior cingulate cortex (ACC) [130] (see also Table 1).

Actions with a higher emotional value evoke stronger responses in the AON of observers than actions with a smaller emotional value [55,57,58,59,61,131,132]. The emotional value may be related to both unpleasant states or events, like fear or anger, and positive or pleasant events, like happiness [58,61,77,131]. The processing of the emotional value of an observed action is closely related to the social functions of the AON [23,25,38,130]. These functions are related, for example, to the activation of a physical readiness to react appropriately to an observed behavior [55,95] or the activation of a state that reflects the emotional state of the observed individual [11,130].

### 3.5. Experiences That Are Accompanied by Conscious Awareness

Motor and somatosensory responses to observed actions in the AON and at the corticospinal and muscular level, as well as emotional reactions, can result in experiences or feelings (of movement, exertion, touch, pain, warmth, cold, threat, or pleasure) that are accompanied by awareness. For a review, see [97]. Experiences or feelings arise in a gradual transition between non-conscious and conscious processing [64,133,134]. Conscious experiences related to observed actions are mainly based on somatosensory and emotional processes [8,11,134,135,136]. Participating somatosensory structures that evoke conscious experiences (secondary somatosensory cortex and insula) interact with motor structures, the activities of which do not in themselves correlate with conscious sensation [134,137]. Sensory, motor, emotional, and motivational information is integrated through the insula, anterior cingulate cortex (ACC), and orbitofrontal cortex (OFC) [11,135]. Together with the amygdala, these structures contribute to conscious, emotionally charged experiences of pictured actions [136]. Such experiences or feelings probably play a central role in the attractiveness that video clips or photos of outdoor activities, sports, fighting, or sex have for many people [102].

## 4. Cognitive Products of Neural Responses to Action Photos in the AON

The AON has evolved as a brain system that processes visual information related to movements of other individuals. Photos are static pictures. Nevertheless, they evoke similar neural responses in the AON as live actions or video clips of actions (Table 1 and Table 2). These responses can be related to different cognitive activities.

Photos of events can generate retinal images similar to events that take place in the real world. Events depicted in photos share a large number of visual stimulus features with the relevant events that took place in real life. Photos are realistic images, yet they only represent a fraction of the multimodal sensory information received by people who observed a real-life event that was photographed. The recording of photos involves a tearing off of information, that is, abstraction, but there are also two significant properties that have been added to the events depicted: duration and interpretation. In photos, changing visual patterns have been given a duration. A depicted event that, when it actually took place, only lasted 1/1000 of a second, can be viewed for any length of time. A photo represents a complex dynamic event through a single image. Viewers of the photo see the visual appearance that the real event conveyed at a certain moment, at a certain point in space, and limited by a certain frame. The meaning that this temporal and spatial extract from the overall information suggests, stands for the entire complex event. Photos allow viewers to gradually “unpack” the information contained in such images and to reconstruct the depicted event in their imagination. In this sense, events that are depicted in photos can be processed and understood through different cognitive activities: rapid perceptual processing, step-by-step conceptual and semantic decoding, or imagery. All of these cognitive activities can be linked to neural activations in the AON of people who are looking at action photos. If action photos convey a similar social perception to seeing live actions, the AON responses to photos must primarily be perceptual processes. In the following, I will briefly discuss the different possible cognitive activities with which AON responses to photos can be related.

**Figure 1:**
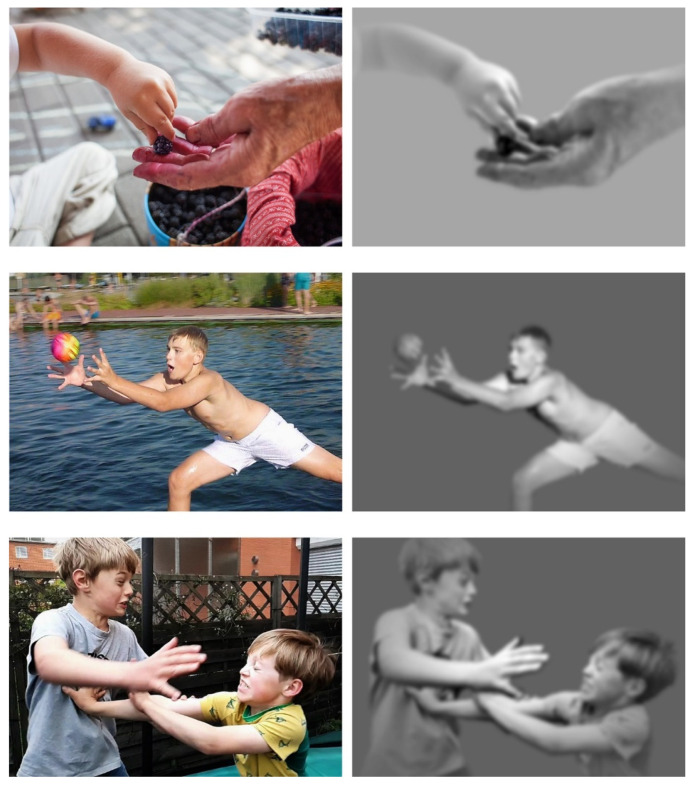
Visual information of action photos that is assumed to be relevant to rapid responses in the action observation network (AON). Note. The picture on the left is the original photo. The modified picture on the right is a hypothetical illustration, of which information from the original photo is relevant with regard to processing in the AON in the early time window up to around 300 milliseconds after picture onset. It has been assumed that the photos were presented to the viewers on their smartphone, which they were holding, and that they had their gaze directed to the display when the photos appeared. The photos had a size of 1505 × 1080 pixels. The viewing angle was 10 × 14°. The spatial resolution of the proposed modifications was based on recent findings on the magnocellular visual pathway [110,111,112,114,115]. The extraction of coarse-scale information during the processing of the picture information in the magnocellular pathway suggests that a distinction between foveal and peripheral vision is not necessary in the first hundreds of ms after picture onset. This assumption is also supported by the analysis of the processing of visual information about moving faces or bodies by Pitcher and Ungerleider [43]. Viewers, however, do not process the entire picture information uniformly but rather select the information in the center of the picture rather than information along its edges [140]. Picture information that is irrelevant to the processing in the AON has been removed in the modifications. They only represent bodies, body parts, faces, and objects that are involved in the actions. All changes to the original photos were made by using Adobe Photoshop (version 21.1.3, Adobe Systems, San Jose, CA, USA). The original color photos were converted into grayscale pictures. Based on studies on magnocellular performance, the modifications had a spatial resolution of 4 cycles per degree [114,115]. The spatial filtering was applied by using a Gaussian blur filter with a 9-pixel kernel for low-pass filtering. In illustrating the center bias of processing, I used a selection mask that corresponded to the “Weight Matrix” in Hayes and Henderson [140] (Figure 2, Panel h). Brightness and contrast were reduced according to the distance to the center of the image. The overall brightness of the modifications corresponded to the original photos. The photos were taken by the author.

Viewers can recognize action photos by associating pictured elements with action-related concepts and integrate these concepts into a representation of an action. The processing of the motor and somatosensory aspects of the concepts involved induces top-down activations in the AON [64,65,119]. In this case, recognizing and understanding action photos would be similar to reading sentences or texts [138]. The interpretation of the recognition and understanding of action photos as a cognitive operation, which is similar to reading, however, is inconsistent with the findings on the specificity and timing of the AON responses to photos. A photo of a boy jumping to catch a ball (as shown in the middle panel of Figure 1) contains a large amount of information that is specific to the boy, his movements, the ball, and the conditions under which the action is performed. The magnocellular system and neurons in the AON of viewers extract and process information relating to the face, hands, body parts, and movement from the entire photo. The findings reviewed so far suggest that a mental representation of the concrete action depicted is established in viewers within 300 ms after the picture’s onset. This representation is fundamentally expressed by visual, motor, and somatosensory neural activation patterns. In order to understand the sentence “the boy jumps to catch the ball,” each individual word must be heard or read and related to the following words. Silently reading the sentence takes about 1.5 s at an average reading speed [139]. The formation of a mental representation that includes the information of the entire sentence may therefore only occur after 1.5 s from the beginning of reading the sentence. This representation is still related to highly generalized action-related information.

Another reason for AON responses to action photos may be that these responses are related to voluntary cognitive operations that occur after the picture has been recognized, based on processing in the ventral visual stream. Such cognitive operations may be motor imagery [68,69,70], mentalizing [64,67], or verbalizations based on semantic representations [3,65,119]. These operations are also associated with neural activities in the AON, but if the AON responses to photos were causally related to these operations, then the responses would occur in a later processing time window after the picture onset. The responses that occur in the time window up to about 300 ms after picture onset are fundamentally related to the processing of the incoming visual information [46,53,66,70,76,105,141].

**Figure 2:**
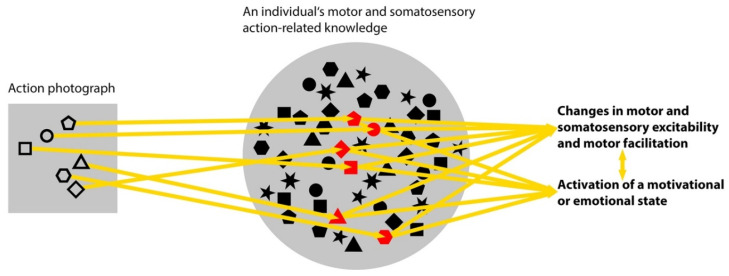
Schematic illustration of the relationship between the representational characteristics of action photos and outcomes of their cognitive processing in the AON. Note. The small, differently shaped objects stand for action elements. These elements relate to movements of body parts, the motor control of muscle activities, somatosensory processes or sensations, action-related objects or contexts, goals, and emotional value. An arrow means “evokes.” The color red signifies “activation”.

The findings on AON responses to specific properties of actions depicted in photos in the early processing time window suggest that these responses are related to perceptual processes [46,53,66,70,76,105,141]. The visual information provided by an action photo has properties that are processed by visual cortical areas as incomplete body-related information (EBA) and movement-related information (MT) (Table 1). EBA and MT then forward the processing output to areas of the AON. Photos provide information that is incomplete compared to live actions. Urgesi and colleagues [56] pointed out that the visual information that people receive from live actions is also often incomplete in natural environments. Moving body parts or objects may be obscured, and obstacles can obstruct the view. For this reason, brain mechanisms have evolved that complete fragmentary movement cues [51,142]. The neural processing of action photos makes use of such mechanisms and generates similar cognitive products as the AON responses to observed live or videoed actions [15]. Figure 2 shows the suggested relationships between the representational characteristics of action photos and the outcomes of their cognitive processing.

## 5. Specific Representational Characteristics of Action Photos

The present review suggests six major representational characteristics of action photos, which influence the strength of the motor and somatosensory neural activations in the AON and the associated corticospinal downstream projections.

### 5.1. Clarity of the Pictured Movements

A clearly recognizable visual representation of movement is difficult to achieve with static pictures [138,143]. In the case of sharp, detailed photos, viewers often cannot recognize whether the depicted individuals or body parts were moving or in a static pose. Motion blur, i.e., the blurred image of a body part or object along its movement trajectory, provides movement-related information, but it does not provide any information about the direction of the movement, and it reduces the clarity of the picture [143,144]. Certain abstract visual patterns, in connection with expansion or rotation, also may suggest movement [142]. A high degree of clarity in the depiction of movement is given when photos provide suggestive clues to the positions that the pictured body parts occupied immediately before and immediately after the moment the photo was taken [22]. Characteristic views of actions support the quick recognition of the course of the action that is immediately following [109].

### 5.2. Visibility of Muscle Activities and Skin

Pictured movements are generated by muscle activities. These activities, muscle contractions, or deformations of the skin can be represented visibly in photos [70]. Muscle activities involve some level of exertion. If greater exertion is depicted, this may elicit stronger reactions in the AON than lower exertion [18].

### 5.3. Visibility of Somatosensory Operations or Sensations

Skin, deformations of skin, muscle contractions, body or hand postures, and facial expressions provide clues about the somatosensory processes involved in an action. The somatic component of the activations that photos evoke in viewers can relate, for example, to touch [36], pain [29,37,39], or interoception [27,32]. The bottom panel of Figure 1 shows an interaction in which somatosensory processing obviously plays a central role. The scuffling boys have their eyes closed to protect them from injury. Their perception and action control are based primarily on information from somatosensory receptors.

### 5.4. Clarity of the Involved Object or Context

The meanings of pictured graspable, familiar objects are presumably also processed in the areas included in Table 1 [98,99,100,145]. In the case of tools, in addition to familiarity with use, elongation may be a decisive property of form with regard to processing in the areas mentioned [98,100]. The meanings of objects, the recognition of which requires complex knowledge of abstract concepts or sociocultural practices (as is the case with medical syringes, bank notes, or hand mixers), are processed in the ventral visual stream [121]. Recognizing the meanings of observed or pictured body movements or somatic sensations often requires the inclusion of information from the environment of an action [44,46,105,146,147]. The action of a woman with the palm of her hand on the cheek of a boy, for example, is made understandable by a bottle of sunscreen on a table next to her. Integrating information about movements of body parts with contextual information requires substantial involvement of conceptual processing and occurs from around 250 ms after picture onset [46,105].

### 5.5. Clarity of the Action Goal

If viewers easily recognize the pictured body parts, their movements, and interactions with another individual or object, they can quickly associate the pictured information with an action goal [52,148]: “the child wants to eat the berry”, “the boy wants to catch the ball”, or “the boys want to push each other down” (Figure 1). Viewers do not have to analyze complex contextual information to ascribe a reason to each of these actions. The association of natural, ambiguous scenes with more complex action goals takes longer and occurs from 250 ms after picture onset [46,105].

Recognizing the goal of a pictured action implies anticipating the final state of the action [91]. Photos that clearly represent movements and the somatosensory processes involved in an action contain more or less predictive information about the achievement or non-achievement of the goal of a pictured action [56]. The point in time at which an action is depicted during its course influences the strength of the reactions in the AON. Pictured actions that have not yet reached their final state evoke stronger activations than completed actions [56].

The goal of an action does not have to be the most important information in a photo. What is most relevant to viewers can also relate to special muscle activities [52], intense exertion [18], somatosensory sensations [29], or the emotional value of bodily activities [32,55,57,58,59]. Understanding the goal pursued by a person slicing a cucumber is probably not a primary processing goal when this person is obviously about to cut their thumb [39] (sample picture in Figure 1).

### 5.6. Emotional Value of the Action or Sensation

Facial expressions [149,150], visible skin [151], and expressive behavior [55,108,152] are effective emotional stimuli [101]. Particularly effective with regards to the strength of the reactions in the AON are photos in which the viewers see actions or sensations that would require them to react quickly if they were to actually perceive the events in the environment [153]. For example, people who would see two boys fighting in a rough and tumble play who might get injured, as shown in the bottom panel of Figure 1, may want to step in and separate the two. Seeing a child grasping a sweet berry to eat would allow observers to remain passive.

Other factors may also influence the emotional value that viewers assign to pictured actions, such as the view from which an action is represented. Viewers may find an action seen from the first-person perspective more relevant than the same action from the third-person perspective [13,29,34,39,53,54,141,154,155,156]. It is unclear, however, whether this is actually the case, because it is not known how abstract or specific the cognitive products are that correlate with the motor and somatosensory activations in the AON [154]. For the same reason, it is still unclear whether the distance from which an action is pictured in a photo modulates the reactions in the AON.

### 5.7. Questions for Future Research

Descriptions of the specific characteristics of action photos, the brain responses they evoke, and the cognitive abilities that such pictures convey are highly speculative at the present time. There is still little knowledge about the properties and processing of action representations in the human brain. There are only a few data on responses of individual neurons to action observation in humans that come from single-unit recording. There are still no data from experiments in which optogenetics was used within the human brain to research the processing of specific action-related representations by neural ensembles [11,84]. TMS combined with EMG and combinations of neuromodulation techniques with non-invasive functional neuroimaging techniques make it possible to attain useful evidence on a number of open questions:

What are the properties of modulations in corticospinal excitability in people who see a photo of an action, compared to modulations in corticospinal excitability in people who observe the same or a similar action while it is actually being performed? What are the time courses of the modulations? Are there similar excitatory or inhibitory effects on the motor activity in both conditions? Is there a similar muscle specificity?

Do visual, motor, and somatosensory neurons in the AON independently categorize elements of complex motor actions depicted in photos taken in natural situations? Do such categorization processes persist if the processing of the visual input through the ventral visual stream is perturbed by the use of TMS or transcranial electrical stimulation (tDCS)?

Does looking at photos activate neural ensembles in viewers that represent specific properties of the concrete actions depicted more comprehensively than through categorization? If so, how high is the degree of specificity that is achieved in the first 150 ms after picture onset, and how high is it after 300 ms? Do the proposed six representational core characteristics of action photos actually correspond to the elements that most strongly influence the strength of the activations in the AON? Is this list complete?

Does the amount of detail in action photos influence the strength of the neural responses in the AON in the first 150 ms after picture onset? Is there an optimal amount of visual details in action photos in order to achieve maximum activation of the AON in the time window of processing of up to 300 ms?

## 6. Conclusions

I reviewed studies that investigated AON responses to pictured actions to examine the assumption that action photos convey special socio-cognitive skills to viewers. Findings from studies that used TMS and EMG, as well as a wide variety of other investigation techniques, suggest that seeing action photos conveys similar processes of social perception as observing live actions. Six representational characteristics of action photos are most relevant in terms of this particular social perception: the clarity of the pictured movements, the visibility of muscle activities and skin, the visibility of somatosensory activities or sensations, the clarity of the involved object or context, the clarity of the action goal, and the emotional value of the pictured action or sensation. People generally use photos for social purposes, such as relating to other people, animals, plants, things, and places or making sense of a complex social world [102]. Viewing action photos enables people to relive what others have done and felt and to prepare their own motor behaviors. These cognitive abilities are based on rapid activations of visual, motor, and somatosensory neurons; activations in brain structures that are involved in processing emotions; as well as specific modulations of excitation states in the body outside the brain.

## Figures and Tables

**Table 1:** Cortical areas and neuroscientific studies in which responses to photos of body movements or actions were observed and which were included in the present review.

Brain Areas	Studies
Extrastriate body area (EBA)	Downing et al., 2006 [14]; Hafri et al., 2017 [15]; Lu et al., 2016 [16]; O’Toole et al., 2014 [17]; Proverbio et al., 2009 [18]; Thierry et al., 2006 [19]
Middle temporal area (MT)	Hafri et al., 2017 [15]; Hermsdörfer et al., 2001 [20]; Kolesar et al., 2017 [21]; Kourtzi & Kanwisher, 2000 [22]; Lu et al., 2016 [16]; O’Toole et al., 2014 [17]; Proverbio et al., 2009 [18]
Additional regions of the posterior superior temporal sulcus (pSTS)	Arioli et al., 2018 [23]; Canessa et al., 2012 [24]; Hafri et al., 2017 [15]; Hermsdörfer et al., 2001 [20]; Kourtzi & Kanwisher, 2000 [22]; O’Toole et al., 2014 [17]; Pierno et al., 2008 [25]; Proverbio et al., 2011 [26]
Inferior parietal lobule (IPL) and/or intraparietal sulus (IPS)	Bühler et al., 2008 [27]; Canessa et al., 2012 [24]; Ferretti et al., 2005 [28]; Gu & Han, 2007 [29]; Hafri et al., 2017 [15]; Hermsdörfer et al., 2001 [20]; Kolesar et al., 2017 [21]; Ogawa & Inui, 2011 [30]; Proverbio et al., 2009 [18]; Redouté et al., 2000 [31]; Wehrum et al., 2013 [32]
Premotor cortex (PMC) and/or inferior frontal gyrus (IFG)	Arioli et al., 2018 [23]; Canessa et al., 2012 [24]; Hafri et al., 2017 [15]; Johnson-Frey et al., 2003 [33]; Kolesar et al., 2017 [21]; Mazzarella et al., 2013 [34]; Ogawa & Inui, 2011 [30]; Pierno et al., 2008 [25]; Proverbio et al., 2009 [18]; Watson et al., 2014 [35]
Primary and/or secondary somatosensory cortex (S1, S2)	Bolognini et al., 2013 [36]; Bühler et al., 2008 [27]; Cheng et al., 2008 [37]; Gu & Han, 2007 [29]; Proverbio et al., 2011 [26]
Insula	Arioli et al., 2018 [23]; Bühler et al., 2008 [27]; Deuse et al., 2016 [38]; Gu & Han, 2007 [29]; Jackson et al., 2005 [39]; Kolesar et al., 2017 [21]; Wehrum et al., 2013 [32]
Anterior cingulate cortex (ACC)	Bühler et al., 2008 [27]; Gu & Han, 2007 [29]; Jackson et al., 2005 [39]; Kolesar et al., 2017 [21]; Proverbio et al., 2009 [18], 2011 [26]; Redouté et al., 2000 [31]; Wehrum et al., 2013 [32]
Orbitofrontal cortex (OFC)	Bühler et al., 2008 [27]; Deuse et al., 2016 [38]; Redouté et al., 2000 [31]; Wehrum et al., 2013 [32]
Amygdala	Deuse et al., 2016 [38]; Ferretti et al., 2005 [28]; Hadjikhani & de Gelder, 2003 [40]; Pierno et al., 2008 [25]; Poyo Solanas et al., 2018 [41]

**Table 2:** Findings on the processing of observed or pictured actions from studies which used transcranial magnetic stimulation (TMS) or methods of non-invasive electrical stimulation.

Changes in Corticospinal Excitability, Motor Facilitation, and/or Downstream Modulation	
Photos as stimuli	Amoruso et al., 2020 [44]; Borgomaneri et al., 2014 [58], 2015 [59], 2015 [55]; Catmur et al., 2011 [60]; Hajcak et al., 2007 [61]; Urgesi et al., 2006 [62], 2010 [56]
Video clips (or apparent motion cues) as stimuli	Mc Cabe et al., 2015 [52]; Ubaldi et al., 2015 [66]; Urgesi et al., 2006 [71]
Real actions as stimuli	Fadiga et al., 1995 [72]; Feurra et al., 2019 [2]
**Muscle specificity in changed motor excitability and facilitation**	
Photos as stimuli	Amoruso et al., 2020 [44]; Borgomaneri et al., 2015 [55]; Catmur et al., 2011 [60]; Urgesi et al., 2006 [62], 2010 [56]
Real actions as stimuli	Fadiga et al., 1995 [72]
**Activations in the AON form a neural simulation of a pictured action**	
Photos as stimuli	Bolognini et al., 2013 [36]; Borgomaneri et al., 2012 [57], 2015 [59]; Urgesi et al., 2006 [62], 2010 [56]
Video clips as stimuli	Bolognini et al., 2011 [54]; Avenanti et al., 2018 [73] ^a^; Jacquet & Avenanti, 2015 [12]
**Specific causal contributions from certain brain areas in action perception**	
Photos as stimuli	Bolognini et al., 2013 [36]; Catmur et al., 2011 [60]; Urgesi et al., 2007 [63]
Video clips as stimuli	Avenanti et al., 2018 [73] ^a^; Bolognini et al., 2011 [54]; Jacquet & Avenanti, 2015 [12]; Valchev et al., 2016 [10], 2017 [74]
**Involvement of somatosensory activations in action perception**	
Photos as stimuli	Bolognini et al., 2013 [36]
Video clips as stimuli	Bolognini et al., 2011 [54]; Jacquet & Avenanti, 2015 [12]; Valchev et al., 2016 [10], 2017 [74]
Real actions as stimuli	Avikainen et al., 2002 [75] ^b^
**Time courses of neural processing stages of pictured actions**	
Photos as stimuli	Avanzini et al., 2013 [64]; Borgomaneri et al., 2014 [58], 2015 [59], 2015 [55]
Video clips (or apparent motion cues) as stimuli	Barchiesi & Cattaneo, 2013 [76]; Ubaldi et al., 2015 [66]
**Close connections between the processing of body postures and emotional value or behavioral relevance**	
Photos as stimuli	Borgomaneri et al., 2012 [57], 2014 [58], 2015 [59], 2015 [55]; Hajcak et al., 2007 [61]; van Loon et al., 2010 [77]

^a^ The method used in this study was transcranial direct current stimulation (tDCS). ^b^ The method used in this study was median nerve stimulation (MNS).
